# Impact of Institutional Expected Practice Guidelines on Oral and Long-acting Intravenous Antibiotic Use for Serious Bacterial Infections

**DOI:** 10.1093/ofid/ofag396

**Published:** 2026-07-01

**Authors:** William Bradford, Joshua Stripling, Jay Olivet, John Grissom, Nomin Bold, Katia Bruxvoort, Russell Griffin

**Affiliations:** Division of Infectious Diseases, Heersink School of Medicine, University of Alabama at Birmingham, Birmingham, Alabama, USA; Division of Infectious Diseases, Heersink School of Medicine, University of Alabama at Birmingham, Birmingham, Alabama, USA; Division of Infectious Diseases, Heersink School of Medicine, University of Alabama at Birmingham, Birmingham, Alabama, USA; UAB Heersink School of Medicine, University of Alabama at Birmingham, Birmingham, Alabama, USA; Division of Infectious Diseases, Heersink School of Medicine, University of Alabama at Birmingham, Birmingham, Alabama, USA; Department of Epidemiology, School of Public Health, University of Alabama at Birmingham, Birmingham, Alabama, USA; Department of Epidemiology, School of Public Health, University of Alabama at Birmingham, Birmingham, Alabama, USA

**Keywords:** antimicrobial stewardship, expected practice, oral antibiotics

## Abstract

**Background:**

Expected practice interventions are a low resource antimicrobial stewardship strategy to standardize prescribing behavior by clinicians. An expected practice intervention to encourage use of oral and long-acting IV (PO/LAIV) antibiotics for serious infections was implemented at a US tertiary care hospital in November of 2022.

**Methods:**

We conducted a quasi-experimental pre/post study to evaluate the effect of guideline release on a primary outcome of length of stay (LOS) and secondary outcomes of inpatient antibiotic days, 3-month readmission, PO/LAIV antibiotic use, microbiologic failure, and adverse drug events. We also assessed key factors associated with use of PO/LAIV therapy receipt.

**Results:**

Among 139 encounters, we found a significant increase in the proportion of encounters in which PO/LAIV therapy was prescribed after the intervention (42.0% vs 60.0%, *P* = .03). On multivariable analysis, LOS did not differ (adjusted rate ratio [aRR] 0.91; 95% CI, .74–1.13), nor did inpatient intravenous antibiotic days (aRR 0.95; 95% CI, .78–1.16). Post-implementation encounters had lower odds of 3-month all cause readmission (adjusted odds ratio [aOR] 0.37; 95% CI, .17–.77) and higher odds of PO/LAIV use at discharge (aOR 2.77; 95% CI, 1.28–5.99).

**Conclusions:**

Expected practice intervention implementation was associated with an increase in PO/LAIV therapy use and a reduction in readmission, supporting its role as a promising, low-resource antimicrobial stewardship strategy warranting further prospective evaluation.

More than 2.8 million antimicrobial-resistant infections occur in the United States each year, resulting in more than 35 000 deaths annually [1]. Antimicrobial stewardship (AMS) has emerged as a key tool in the fight against resistant infections [2–4]. However, many conventional AMS approaches, such as prospective audit and feedback and clinical decision support, are resource-intensive to implement, especially in large health systems, and can limit clinicians’ perceived autonomy [3, 4]. One low cost, potentially high impact approach to AMS that has emerged in recent years is the development of expected practice guidelines [2]. In this approach, a guideline document detailing antimicrobial use is developed and released at an institution level; this document sets a general expectation of medical practice by clinicians. Typically developed by guideline committees comprised of subspecialists, anticipated end users, and other stakeholders, the intent of the document is to establish a local standard of care to improve clinician comfort and adherence with a given evidence-based practice approach [2, 4]. Expected practice and related approaches like clinical pathways are associated with significant improvements in patient-centered outcomes across a variety of domains, but they remain understudied and potentially underutilized in AMS [2, 5–7]. By outlining a clear institutional standard of care, expected practice differs from other low-cost/low-barrier AMS approaches like prescription guidance or antimicrobial timeouts by giving institutional authority to best practice antimicrobial prescribing [2, 4].

One key focus of AMS has been reducing prescribing of traditional intravenous (IV) antimicrobials in favor of oral (PO) and long-acting IV (LAIV) options. Evidence has mounted across multiple disease states that highly orally bioavailable PO and LAIV antibiotics are equivalent or superior to traditional IV treatments for most applications [8–11]. The emergence of PO and LAIV is particularly valuable for patients for whom traditional outpatient parenteral antibiotic therapy (OPAT) is not a feasible option, including people experiencing homelessness (PEH) and people who use drugs (PWUD), who are traditionally excluded from OPAT programs based on the widely held belief that long-term parenteral access is unsafe in these groups [12, 13]. However, despite decades of evidence demonstrating safety and efficacy, one major clinician-level barrier to making this switch from traditional IV therapies toward PO and LAIV is clinician concern about institutional standard of care for serious infections [14–17]. To this end, the University of Alabama Birmingham (UAB) Antimicrobial Stewardship Committee released a set of expected practice guidelines in November 2022 that outlined specific recommendations for transitioning therapy from conventional IV to PO and LAIV therapy options (Supplementary Material 1). These expected practice guidelines were designed to be used by clinicians on the inpatient infectious diseases consult service with the intent of reducing IV antibiotic utilization and improving treatment for PO/LAIV eligible infections. They were publicized via departmental email and presentation in various faculty meetings, but their use was not enforced or monitored in any way after release. These guidelines’ effectiveness in achieving their stated goal of increasing transition to PO/LAIV therapy on hospital discharge and reducing inpatient expenditures is unknown.

In this study, our primary aim was to evaluate the performance of these expected practice guidelines in obtaining their stated objective of reducing inpatient resource utilization, operationalized via a primary outcome of length of stay (LOS). Secondarily, we sought to identify patient-level characteristics associated with the receipt of PO/LAIV therapy. We hypothesized that there would be a significant increase in utilization of PO/LAIV therapy and reduction in LOS and readmission after guideline release among patients with serious infections (to whom the guideline applied) and that the safety profiles and microbiologic failure rates before versus after the guideline release would be similar. We furthermore hypothesized that PO/LAIV therapy receipt over the entire study period would be more common in PEH and PWUD.

## METHODS

### Development and Implementation of the Expected Practice Guidelines

The UAB Antimicrobial Stewardship Committee identified the need for an intervention to empower providers to shift toward PO/LAIV therapies during the COVID-19 pandemic, when routine OPAT and inpatient IV antibiotic administration for the duration of serious infections became impracticable due to intensive resource pressure for inpatient beds imposed by the COVID-19 pandemic. The committee convened a group of infectious diseases (ID) clinicians, including physicians, advance practice prescribers, and pharmacists, along with general inpatient pharmacists and leaders within the pharmacy and therapeutics committee, to develop a set of guidelines. The chair of the committee was an ID physician. The guidelines were developed using a committee-based approach involving an intensive, dedicated, scoping review of the relevant literature before ultimately being approved by the UAB Pharmacy and Therapeutics Committee on 22 November 2022. They were immediately released for use in practice after approval and were publicized by presentation at ID faculty meetings, distribution via email, and discussion by ID pharmacists during ID consult rounds.

### Study Design and Setting

The UAB Health System is a large tertiary care hospital system located in the southeastern United States. Comprised of 1396 staffed beds across two campuses, the institution has full subspecialty coverage including 6 infectious diseases consult services and a large OPAT program [18]. As a safety net hospital for the region, UAB sees a disproportionate number of underserved and uninsured patients [19]. At UAB, the ID consult team sees patients and makes recommendations on antimicrobial therapy and is primarily responsible for IV and long-acting IV antimicrobial prescribing on discharge, while the primary team has responsibility for oral antimicrobial prescribing. Departures from ID consult service-recommended antimicrobial prescribing are extremely rare.

We performed a quasi-experimental pre/post study to assess the effect of expected practice guideline release on a primary outcome of LOS at UAB Hospitals. We identified all encounters in a six-year period between January of 2018 and October of 2024 that (1) had an ID consult, (2) involved an accompanying encounter-associated ICD-10 code corresponding to one of the serious infection states of interest (Supplementary Material 2; native valve endocarditis, bacteremia, osteomyelitis, and joint infection), and (3) involved an adult patient aged ≥18 years. From these encounters, we randomly selected 75 encounters from the pre-intervention period and 75 encounters from the post-intervention period. Originally, 100 encounters from each period had been planned based on power and sample size calculations to detect a difference in LOS of 2 days at 80% power with a two-sided alpha of 0.05; however, after an interim analysis at N = 150 identified nearly identical lengths of stay between the two groups (median 7.9 vs 8.4 days, *P* = .95), further abstraction was halted as no further power benefit was anticipated with additional review.

The study leader manually adjudicated charts to ensure that they met inclusion criteria of (1) presence of one of the diagnoses of interest treated by the infectious diseases consult team during the encounter and (2) infection involving one of the organisms specified in the expected practice guidelines (Supplementary Material 1) with a PO/LAIV option available based on available chart information, and (3) survival in hospital long enough to complete appropriate antibiotic therapy. Patient-directed discharge was not considered an exclusion criterion. We included those who met these criteria for primary analysis. Quality assurance (QA) was performed during adjudication, and any patients who were found not to have met inclusion criteria on detailed chart abstraction were excluded from the cohort at that point. We did not perform any type of check to assess whether providers adhered to the guidelines in treatment. We used REDCap tools hosted at UAB to manage study data [20]. This study was approved by the Institutional Review Board (IRB-300013678).

### Exposure and Outcome Definitions

We defined the primary outcome of LOS as days between admission and discharge. Pre-specified secondary outcomes of interest were overall use of PO/LAIV therapy, incidence of antibiotic-related adverse events, three-month readmission, microbiologic failure, and total days of inpatient IV antibiotics received. Of note, disease state-specific definitions of microbiologic failure were used for each of the infection types (definition for each infection type in Supplementary Table 1). Elixhauser Comorbidity Index was calculated using the ICD-10 code-based approach outlined by the Agency for Healthcare Research and Quality (AHRQ) [21]. Data were abstracted using standardized forms with ID physician adjudication. Only organisms that were identified as being true pathogens contributing to infection were included in abstraction. Colonizing/contaminant organisms were not included. Colonization and contamination were determined on the judgement of the chart abstraction team, which relied on clinical notes and treatment decisions to treat or not treat a given organism. Patients without a documented inpatient admission within the UAB health system within 90 days of discharge were assumed to have no readmission; we were unable to capture readmissions occurring at outside institutions. All abstraction was performed using REDCap forms (Supplementary Material 3). All variable operationalizations and sources are available in Supplementary Table 2.

### Statistical Analysis

We used descriptive statistics as appropriate to summarize data. Missing data were treated as missing at random; complete case analysis was used for all analyses. For bivariate analyses, we compared categorical variables using a Fisher's exact test or chi-square, while continuous variables were compared using a t-test, analysis of variance, or nonparametric alternative (Wilcoxon Rank Sums). Treating use of PO/LAIV as the outcome of interest, we assessed the independent effect of age, race, sex, homelessness, injection drug use, and Elixhauser comorbidity count as exposures. We assessed multicollinearity using Pearson correlation coefficients and did not identify any problematic collinearity (defined as |R|>0.80).

For the primary outcome of length of stay (LOS), a multivariable negative binomial regression model with a log link was used to estimate rate ratios (RRs) and 95% confidence intervals (CIs) comparing encounters before versus after guideline implementation. Secondary outcomes were analyzed using separate multivariable regression models: inpatient intravenous antibiotic days were modeled using negative binomial regression, while 3-month readmission and discharge use of PO/LAIV antibiotics were modeled using logistic regression to estimate adjusted odds ratios (aORs), adjusted rate ratios (aRRs), and 95% CIs. Covariates were prespecified based on clinical relevance and potential confounding and included age, race, sex, housing status, insurance status, and Elixhauser comorbidity burden. Generative artificial intelligence tools were used to assist with statistical code drafting and syntax refinement; all modeling decisions, code implementation, and verification of results were performed directly by the investigators. All analyses were conducted using SAS version 9.4 (Cary, NC).

## RESULTS

### Cohort Characteristics

A total of 12 907 encounters were identified by initial ICD-10 codes and ID consult-based identification. A sample of 261 encounters were reviewed, and 150 met inclusion criteria after initial chart review (Table 1). After quality assurance (QA) review to ensure inclusion and exclusion criteria were met (leading to 9 exclusions, mainly on the basis of non-qualifying infection type) and a post hoc decision to exclude two observations involving very long lengths of stay (>60 days), 11 additional patients were excluded for a total of 139 patients included in the analysis. Most patients were middle-aged (mean age 53.3, SD 16.8), male (n = 74, 53.2%), white (n = 85, 61.2%), and publicly insured (n = 88, 63.3%). The median Elixhauser comorbidity index (ECI) was 4 (IQR 2–6). The most common type of serious infection was bacteremia (n = 84, 60.4%) followed by osteomyelitis (n = 54, 38.8%). Septic arthritis (n = 16, 11.5%) and native valve endocarditis (n = 6, 4.3%) were relatively less common. Multiple infection types were identified in 21 patients (15.1%) The most common organism identified was *Staphylococcus aureus* (n = 56, 40.3%) followed by other (n = 49, 35.3%) and gram-negative organisms (n = 45, 32.4%). The most common discharge antibiotic regimen was PO (n = 68, 48.9%) followed closely by traditional IV (n = 67, 48.2%). A total of 16 patients (11.5%) were discharged on more than one type of antibiotic therapy. The median LOS was 8.3 days (IQR 5.7–15.1). Median inpatient antibiotic days were 8.0 (IQR 6.0–13.0), and readmission occurred within 3 months of discharge in 50 patients (36.0%). Microbiologic failure occurred in 9 (6.5%) and adverse drug events leading to antimicrobial discontinuation occurred in 5 (3.9%).

**Table 1.** Sample Characteristics Stratified by Exposure of Time Period (N = 139)

Sample Characteristic	Overall (N = 139)	Before 11/22/2022 (n = 69)	On Or After 11/22/2022 (n = 70)	*P V*alue
**Demographics**	…	…	…	
Age, mean (SD)	53.3 (16.8)	52.6 (17.5)	54.0 (16.1)	.61^a^
Male, n (%)	74 (53.2)	39 (56.5)	35 (50.0)	.44^b^
Race, n (%)	…	…	…	
White	85 (61.2)	44 (63.8)	41 (58.6)	.53^b^
Black	61 (36.7)	23 (33.3)	28 (40.0)	.41^b^
Other	3 (2.2)	2 (2.9)	1 (1.40)	.62^c^
**Social history**	…	…	…	
Homelessness, n (%)	6 (4.3)	3 (4.3)	3 (4.3)	1.00^c^
Insurance status, n (%)	…	…	…	
Public	88 (63.3)	44 (63.8)	44 (62.9)	.91^b^
Private	43 (30.9)	20 (29.0)	23 (32.9)	.62^b^
Uninsured	8 (5.8)	5 (7.2)	3 (4.3)	.49^c^
Injection drug use, n (%)	9 (6.5)	4 (5.8)	5 (7.1)	1.00^c^
Illicit drug use, n (%)	…	…	…	.74^b^
Current	23 (16.5)	11 (15.9)	12 (17.1)	
Past	19 (13.7)	8 (11.6)	11 (15.7)	
Never	97 (69.8)	50 (72.5)	47 (67.1)	
**Medical history**	…	…	…	
Elixhauser CI, median (IQR)	4 (2, 6)	4 (2, 6)	4 (3, 6)	.30^d^
Elixhauser comorbidities, n (%)	…	…	…	.78^b^
0–1	23 (16.5)	10 (14.3)	13 (18.8)	
2–4	60 (43.2)	31 (44.9)	29 (41.4)	
5–8	47 (33.8)	21 (30.4)	26 (37.1)	
9 or more	9 (6.5)	4 (5.8)	5 (7.1)	
Infection type, n (%)	…	…	…	
Bacteremia	84 (60.4)	46 (66.7)	38 (54.3)	.53^b^
NVE	6 (4.3)	4 (5.8)	2 (2.9)	.44^c^
Osteomyelitis	54 (38.8)	25 (36.2)	29 (41.4)	.53^b^
Septic arthritis	16 (11.5)	6 (8.7)	10 (14.3)	.30^b^
Hardware involvement, n (%)	28 (20.1)	14 (20.3)	14 (20.0)	.97^b^
Organism, n (%)	…	…	…	
*Staphylococcus aureus*	56 (40.3)	32 (46.4)	24 (34.3)	.15^b^
Gram negative organism	45 (32.4)	23 (33.3)	22 (31.4)	.81^b^
*Streptococcus* spp.	20 (14.4)	4 (5.8)	16 (22.9)	.**004**^b^
*Enterococcu*s spp.	15 (10.8)	6 (8.7)	9 (12.9)	.43^b^
Other^e^	49 (35.3)	19 (27.5)	30 (42.9)	.059^b^

Abbreviations: CI, Comorbidity index; IQR, Interquartile range; NVE, Native valve endocarditis; SD, standard deviation.

^a^t-test.

^b^Chi-square test.

^c^Fisher's exact test.

^d^Wilcoxon rank-sum test.

^e^“Other” organisms included mainly non-aureus Staphylococci and *Corynebacterium* spp.

### Bivariate Analysis

Patients were similar in their baseline sociodemographic characteristics across the pre- versus post-intervention periods, with the typical patient being older (Table 1; average age 52.6 vs 54.0, *P* = .61), white (63.8% vs 58.6%, *P* = .53), male (56.5% vs 50.0%, *P* = .53), housed (95.7% vs 95.7%, *P* = .99), and publicly insured (63.8% vs 62.9%, *P* = .91). Infection types were also similar across the study periods, with most patients having bacteremia (66.7% vs 54.3%, *P* = .14) and/or osteomyelitis (36.2% vs 41.4%, *P* = .53). *S. aureus* was the most frequently identified organism in both time periods (46.4% vs 34.3%, *P* = .15). *Streptococcus* species were less common in the pre-intervention period (5.8% vs 22.9%, *P* = .004). Polymicrobial infections were also less common pre- compared to post-intervention (14.5% vs 28.5%, *P* = .04). PO and/or LAIV antimicrobial therapy was more frequently used in the post- versus the pre-intervention period (Table 2; 60.0% vs 42.0%, *P* = .034). PO antimicrobials were less likely to be prescribed in the pre- versus the post-intervention period (39.1% vs 58.6%, *P* = .02), while traditional IV antimicrobials were concordantly more likely to be prescribed on discharge pre- compared to post-intervention (62.3% vs 34.3%, *P* = .0009). Adverse drug events were rare in both time periods, and rates were similar (5.8% vs 1.4%, *P* = .21) within power limitations. Microbiologic failure was also rare but similarly common across time periods (5.8% vs 7.1%, *P* = .75). Average LOS was similar (median 7.9 vs 8.4 days, *P* = .95), as were inpatient antibiotic days (median 8.0 vs 8.0, *P* = .86), while 3-month readmission was less common in the post- compared to the pre-guideline period (46.4% vs 25.7%, *P* = .01).

**Table 2.** Outcomes Stratified by Time Period (N = 139)

Outcome	Overall (N = 139)	Before 11/22/2022 (n = 69)	After 11/22/2022 (n = 70)	*P V*alue
LOS, median (IQR)	8.3 (5.7, 15.1)	7.9 (5.7, 15.1)	8.4 (5.6, 15.5)	.95^a^
3-m readmission, n (%)	50 (36.0)	32 (46.4)	18 (25.7)	.**01**^b^
Inpatient IV antibiotic days, median (IQR)	8.0 (6.0, 12.5)	8.0 (6.0, 12.5)	8.0 (6.0, 13.0)	.86^a^
Discharge antibiotic regimen, n (%)	…	…	…	
PO and/or LAIV	71 (51.1)	29 (42.0)	42 (60.0)	.**034**^b^
PO	68 (48.9)	27 (39.1)	41 (58.6)	.**02**^b^
Long-acting IV	6 (4.3)	5 (7.2)	1 (1.4)	.12^c^
IV	67 (48.2)	43 (62.3)	24 (34.3)	**<**.**001**^b^
Other	14 (10.1)	7 (10.1)	7 (10.0)	.98^b^
Adverse antibiotic events, n (%)	5 (3.6)	4 (5.8)	1 (1.4)	.21^c^
Microbiologic failure, n (%)	9 (6.5)	4 (5.8)	5 (7.1)	1.00^c^

Abbreviations: AKI, acute kidney injury; IQR, Interquartile range; IV, intravenous; LOS, length of stay; PO, per os.

^a^Wilcoxon rank-sum test.

^b^Chi-square test.

^c^Fisher's exact test.

### Multivariable Analysis

In multivariable regression analyses adjusting for age, race, sex, housing status, insurance status, and Elixhauser comorbidity burden (Table 3), expected practice guideline implementation was not associated with a significant change in the primary outcome of LOS (adjusted rate ratio [aRR] 0.91; 95% CI, .74–1.13). Similarly, no significant difference was observed in the secondary outcome of inpatient intravenous antibiotic days (aRR 0.95; 95% CI, .78–1.16). In contrast, encounters occurring after guideline release were associated with lower odds of 3-month readmission (adjusted odds ratio [aOR] 0.37; 95% CI, .17–.77). Post-implementation encounters were also more likely to receive PO/LAIV therapy at discharge (aOR 2.77; 95% CI 1.28–5.99) compared to pre-implementation encounters.

**Table 3.** Adjusted Associations Between Expected Practice Implementation (Post vs Pre) and Outcome of Interest

Outcome	Distribution (Linking Function)	Adjusted Effect^a,b^ (95% CI)	*P V*alue
**Primary outcome**			
LOS	Negative binomial (log)	0.91 (0.74–1.13)	.39
**Secondary outcomes**			
Inpatient IV antibiotic days	Negative binomial (log)	0.95 (0.78–1.16)	.62
3-m readmission	Binomial (logit)	0.37 (0.17, 0.77)	.**008**
PO/LAIV use on discharge	Binomial (logit)	2.77 (1.28–5.99)	.**01**

Abbreviations: CI, confidence interval; LAIV:, long-acting intravenous; LOS, length of stay; PO, by mouth; RR, rate ratio.

^a^Adjusted odds ratio (3-m readmission, PO/LAIV use) or adjusted rate ratio (LOS, inpatient antibiotic days).

^b^Adjusting for age, race (white vs non-white), sex, housing status, insurance status (public vs non-public), and number of Elixhauser comorbidities (0–1, 2–4, 5–8, or 9 or more).

### Associations With PO/LAIV Therapy

Compared to patients who did not receive PO/LAIV therapy, those who did receive PO/LAIV therapy were more likely to be younger (Supplementary Table 3; 49.3 vs 57.5, *P* = .003), non-white (49.3% vs 28.9%, *P* = .01), and have fewer Elixhauser medical comorbidities (median 3 vs 5, *P* = .002). In terms of infection-specific characteristics, those who did not receive PO/LAIV therapy compared to those who did were less likely to have *S. aureus* infection (51.5% vs 29.6%, *P* = .009), more likely to have a gram negative infection (45.1% vs 19.1%, *P* = .001), less likely to have bacteremia (43.7% vs 77.9%, *P* < .001), and more likely to have osteomyelitis (49.3% vs 27.9%, *P* = .01) compared to other infection types. Those who did not receive PO/LAIV antibiotics were significantly less likely to have microbiologic treatment failure (Supplementary Table 4; 1.5% vs 11.3%, *P* = .03). Rates of adverse drug events (4.4% vs 2.8%, *P* = .68), hardware involvement (17.6% vs 22.5%, *P* = .47), current illicit drug use (11.8% vs 21.1%, *P* = .22), injection drug use (5.9% vs 7.0%, *P* = 1.00), and public insurance (63.2% vs 63.4%, *P* = .99) did not differ significantly between the two groups. Using PO/LAIV therapy as the outcome variable in multivariable logistic regression, non-white race was significantly associated with use of PO/LAIV therapy in the fully adjusted model (Table 4; adjusted odds ratio [aOR] 0.35, 95% CI .16 to .79), while other associations were non-significant.

**Table 4.** Results of Multivariable Logistic Regression With Outcome of Use of PO or LAIV Therapy

Exposure	aOR (95% CI)
Age	0.98 (0.96, 1.01)
Race (ref = white)	**0.35** (**0.16, 0.79)**
Sex (ref = male)	1.29 (0.61, 2.72)
Homelessness (ref = housed)	0.54 (0.07, 4.39)
Any IDU (ref = no use)	1.86 (0.29, 12.03)
Elixhauser comorbidity count (ref = 0–1 comorbidities)	…
2–4 comorbidities	0.89 (0.29, 2.66)
5–8 comorbidities	0.32 (0.10, 1.05)
9 or more comorbidities	0.19 (0.03, 1.33)

Abbreviations: aOR, adjusted odds ratio; CI, confidence interval; Ref, reference.

## DISCUSSION

In this retrospective observational study, we identified a significant increase in utilization of PO/LAIV antibiotics and a significant reduction in inpatient readmissions after an expected practice guideline rollout. However, we did not identify any significant differences in other health system cost-sensitive metrics of interest, including our primary outcome of LOS and our secondary outcome of inpatient antibiotic days. Patients who received PO/LAIV therapy were more likely to be younger, non-white, less medically complex, and have bacteremia or osteomyelitis compared to those who did not receive PO/LAIV therapy. Concerningly, there were significantly more episodes of microbiologic treatment failure in the PO/LAIV treatment group than the standard therapy group, but there were no detectable differences in adverse drug events.

The finding that expected practice guidelines were associated with a significant increase in PO/LAIV antibiotic use and reduction in all cause readmission is consistent with prior studies involving similar interventions and suggests that this approach is promising as a low-barrier, low-cost approach to change clinician prescribing behavior [5, 8]. The increased proportion of microbiologic treatment failures identified in patients who received PO/LAIV therapy, while likely attributable to confounding by indication due to nonrandom patient selection, represented a concerning trend that deserves further attention in a future, better-powered analysis. A posthoc review of these cases identified inadequate source control as a key contributor in 7 of the 8 antimicrobial failures, but further study would be helpful to further evaluate this finding. Such a finding would reflect an important efficacy-effectiveness gap as randomized prospective efficacy studies involving PO/LAIV therapy did not demonstrate any similar safety concerns [8, 22, 23]. The other safety endpoint of adverse drug events was underpowered here but did not demonstrate any similar concerning trends. Overall, we believe these findings are consistent with existing evidence on expected practice approaches indicating that expected practice is a safe, low barrier tool to change clinician behavior change at a health system level and that PO/LAIV therapies are fundamentally safe treatments.

Effective AMS is critical to prevent the rising tide of antimicrobial resistance, but most approaches are time intensive, expensive, and frequently face pushback when interfacing with clinicians [1, 3, 4, 7]. In contrast, expected practice interventions represent a low-cost, low-barrier strategy that may achieve comparable improvements in prescribing while fostering clinician autonomy. We found increased use of PO/LAIV therapy and reduced readmissions in patients with serious infections following guideline rollout, which has the potential address this serious implementation gap. The rare use of LAIV therapy (4.3% overall) in our cohort likely reflects limited institutional familiarity with dalbavancin and the logistical and cost barriers associated with its administration relative to oral alternatives, rather than a lack of eligible patients. The potential signal toward increased microbiologic failures underscores the need for circumspect and measured implementation. In an increasingly resource-constrained implementation environment, expected practice may represent a pragmatic complement to more labor-intensive approaches and warrants continued study in larger, prospective evaluations.

One major limitation is that our study used a retrospective observational design and is subject to potential residual confounding. Although we adjusted for known confounders (housing status, comorbidity burden, race), there were likely unmeasured differences (ie, confounding by indication) in characteristics of patients who received PO/LAIV therapy, especially since the use of guidelines was not monitored or enforced. It is well known that PO/LAIV therapies are used as alternative therapies in patients in whom “best practice” or “usual therapy” (ie, traditional IV antibiotics) are either not feasible or not practical [12, 13]. We also did not incorporate a washout period to allow time for adoption of the intervention; doing so may have led to an increase in the observed effects of the intervention. We did not formally assess guideline adherence by agent selection or dosing; had we done so, restriction to guideline-concordant encounters may have attenuated the observed association between PO/LAIV therapy and microbiologic failure, as non-adherent regimens could have contributed to this signal. Additionally, unrelated temporal trends not accounted for in our model (eg, COVID-related changes in readmission) may have accounted for some of the large effect size observed in readmissions. A prospective evaluation of an expected practice intervention, ideally a randomized controlled trial (RCT), would be the ideal approach to establish the independent effect of an expected practice intervention.

In summary, this study found that implementation of an expected practice intervention focused on prescribing PO/LAIV therapy was associated with a meaningful increase in PO/LAIV therapy use and an accompanying reduction in readmissions at one large tertiary care hospital. However, there was no associated reduction in LOS or inpatient IV antibiotic days, two important health system cost-sensitive indicators. These findings support existing evidence that expected practice can be a valuable tool in antimicrobial stewardship that has the potential to positively influence clinician prescribing behavior in a low-barrier, low-cost manner.

## Supplementary Material

ofag396_Supplementary_Data

## Supplementary Data

Supplementary materials are available at *Open Forum Infectious Diseases* online. Consisting of data provided by the authors to benefit the reader, the posted materials are not copyedited and are the sole responsibility of the authors, so questions or comments should be addressed to the corresponding author.
