# Supplementary material for: Impact of Institutional Expected Practice Guidelines on Oral and Long-acting Intravenous Antibiotic Use for Serious Bacterial Infections
Source: Open Forum Infect Dis. 2026 Jul 1;13(7):ofag396. doi: 10.1093/ofid/ofag396 (PMC13348848; doi:10.1093/ofid/ofag396)
Supplement: ofag396_Supplementary_Data [file ofag396_supplementary_data.pdf]

Supplemental Material

Supplemental Table 1. Disease state-specific definitions of microbiologic failure.

Infection Type	Definition of Microbiologic Failure
**Bacteremia**	Growth of at least one index organism (not necessarily with identical susceptibilities) from blood culture within 30 days of completion of prescribed antibiotic therapy, as documented in the UAB microbiology records or infectious diseases consult documentation from subsequent encounters.
**Osteomyelitis**	Isolation of at least one index organism (not necessarily with identical susceptibilities) from a sterile site or sinus tract culture obtained from the same anatomic site within 90 days of completion of prescribed antibiotic therapy, as documented in UAB microbiology records or infectious diseases consult documentation from subsequent encounters.
**Septic Arthritis**	Isolation of at least one index organism (not necessarily with identical susceptibilities) from a sterile site or sinus tract culture obtained from the same anatomic site within 90 days of completion of prescribed antibiotic therapy, as documented in UAB microbiology records or infectious diseases consult documentation from subsequent encounters.
**Native Valve Endocarditis**	Isolation of at least one index organism (not necessarily with identical susceptibilities) from a sterile site or sinus tract culture obtained from the same anatomic site within 90 days of completion of prescribed antibiotic therapy, as documented in UAB microbiology records or infectious diseases consult documentation from subsequent encounters.

Supplemental Table 2. Exposure and outcome variable operationalizations.

Variable	Operationalization
**Age**	Patient age at encounter as recorded in EHR.
**Sex**	Patient sex as recorded in EHR demographics.
**Race**	Patient race as recorded in EHR demographics. In case of missing data from demographics, this data was abstracted from clinical documentation.
**Date of admission**	Date that patient was admitted to the hospital as recorded in EHR.
**Housing status**	Patient housing status as recorded in EHR demographics or social work documentation.
**Discharge disposition**	Patient discharge disposition as recorded in provider and social work documentation.
**Insurance status**	Patient insurance status as recorded in EHR.
**Elixhauser comorbidity index**	Components of the Elixhauser Comorbidity Index (history of certain medical comorbidities) as recorded in encounter-specific ICD codes.
**Infection type**	Type of serious bacterial infection diagnosed during encounter as established by review of infectious diseases team documentation.
**Organism**	Microbial organism causing infection, identified by review of provider documentation and microbiological reports. Colonizing microorganisms were not included.
**Hardware involvement**	Yes/no response of whether medical hardware was involved in the infection, as determined by review of infectious diseases team and other relevant documentation.
**Substance use history**	Information on substance use history including specific substances used, route of substance use (e.g. IV versus insufflation), and other parameters relevant to substance use history as recorded in relevant provider documentation. Past substance use was defined as any mention of "history of" illicit drug use during encounter documentation.
**IV Antibiotic Duration**	Duration of inpatient IV antibiotics received as recorded in medication administration record.
**Psychiatric illness history**	Information on psychiatric illnesses including specific diagnoses, dates diagnoses were noted, and relevant treatments as recorded in relevant provider notes.
**Antibiotic-related adverse events**	Adverse events related to antibiotic therapy including acute kidney injury, drug discontinuation for adverse event, vascular access-related complication, and mortality during duration of antimicrobial treatment.
**Readmission date**	Date of first and subsequent readmissions after presentation for 3 months after index admission.
**Post-discharge antibiotic treatment**	Antibiotic treatment type prescribed on discharge, as established by review of infectious diseases and general provider documentation.
**Length of stay**	Length of stay during a hospital encounter, as recorded in EHR.

Supplemental Table 3. Sample characteristics stratified by exposure of use of PO/LAIV therapy (N=139).

**Sample characteristic**	**Overall (N=139)**	**No use of PO/LAIV therapy (n=68)**	**Use of PO/LAIV therapy (n=71)**	***p*-value**
**Demographics**				
Age, mean (SD)	53.3 (16.8)	57.5 (17.3)	49.3 (15.3)	**0.003**[1]
Male, n(%)	74 (53.2)	32 (47.1)	42 (59.2)	0.15[2]
Race, n(%)				
White	85 (61.2)	49 (71.1)	36 (50.7)	**0.01**[2]
Black	61 (36.7)	19 (27.9)	32 (45.1)	**0.04**[2]
Other	3 (2.2)	0 (0.0)	3 (4.2)	0.24[3]
**Social history**				
Homelessness, n(%)	6 (4.3)	3 (4.4)	3 (4.2)	1.00[3]
Insurance status, n(%)				
Public	88 (63.3)	43 (63.2)	45 (63.4)	0.99[2]
Private	43 (30.9)	21 (30.9)	22 (31.0)	0.99[2]
Uninsured	8 (5.8)	4 (5.9)	4 (5.6)	1.00[3]
Injection drug use, n(%)	9 (6.5)	4 (5.9)	5 (7.0)	1.00[3]
Illicit drug use, n(%)				0.22[2]
Current	23 (16.5)	8 (11.8)	15 (21.1)	
Past	19 (13.7)	8 (11.8)	11 (15.5)	
Never	97 (69.8)	52 (76.5)	45 (63.4)	
**Medical History**				
Elixhauser CI, median (IQR)	4 (2, 6)	5 (3, 6)	3 (2, 5)	**0.002**[4]
Elixhauser comorbidities, n (%)				**0.02**[2]
0-1	23 (16.5)	8 (11.8)	15 (21.1)	
2-4	60 (43.2)	24 (35.3)	36 (50.7)	
5-8	47 (33.8)	29 (42.6)	18 (25.4)	
9 or more	9 (6.5)	7 (10.3)	2 (2.8)	
Infection type, n(%)				
Bacteremia	84 (60.4)	53 (77.9)	31 (43.7)	**<0.001**[2]
NVE	6 (4.3)	5 (7.4)	1 (1.4)	0.11[3]
Osteomyelitis	54 (38.8)	19 (27.9)	35 (49.3)	**0.01**[2]
Septic arthritis	16 (11.5)	6 (8.8)	10 (14.1)	0.33[2]
Hardware involvement, n(%)	28 (20.1)	12 (17.6)	16 (22.5)	0.47[2]
Organism, n(%)				
Staphylococcus aureus	56 (40.3)	35 (51.5)	21 (29.6)	**0.009**[2]
Gram negative organism	45 (32.4)	13 (19.1)	32 (45.1)	**0.001**[2]
Streptococcus spp.	20 (14.4)	9 (13.2)	11 (15.5)	0.70[2]
Enterococcus spp.	15 (10.8)	5 (7.4)	10 (14.1)	0.20[2]
Other*	49 (35.3)	20 (29.4)	29 (40.8)	0.16[2]

* "Other" organisms included mainly non-aureus Staphylococci and Corynebacterium spp.
Abbreviations: CI: Comorbidity index; IQR: Interquartile range; NVE: Native valve endocarditis; SD: Standard deviation
[1] t-test
[2] chi-square test
[3] Fisher's Exact test
[4] Wilcoxon rank-sum test

Supplemental Table 4. Outcomes stratified by exposure of use of PO/LAIV therapy (N=139).

**Outcome**	**Overall (N=139)**	**No use of PO/LAIV therapy (n=68)**	**Use of PO/LAIV therapy (n=71)**	***p*-value**
LOS, median (IQR)	8.3 (5.7, 15.1)	8.7 (5.7, 15.6)	7.9 (5.7, 12.9)	0.55[1]
Readmission	50 (36.0)	24 (35.3)	26 (36.6)	0.87[2]
Inpt IV antibiotic days, median (IQR)	8.0 (6.0, 12.5)	9 (6, 13)	7.5 (6, 13)	0.11[1]
Adverse antibiotic events, n(%)	5 (3.6)	3 (4.4)	2 (2.8)	0.68[3]
Microbiologic failure, n(%)	9 (6.5)	1 (1.5)	8 (11.3)	**0.034[3]**

Abbreviations: AKI: acute kidney injury; IQR: Interquartile range; IV: intravenous; LOS: Length of stay; PO: per os

[1] Wilcoxon rank-sum test

[2] chi-square test

[3] Fisher's Exact test

Supplemental Material 1: PO/LAIV guidelines

UAB MEDICINE *Clinical Interdisciplinary Guidelines*

Author:	Erin Yarbrough	8/22/22	Pages 1 of	3
	AVP, Clinical Operations	Date	Written:	8/22/22
Endorsed:	Antimicrobial Stewardship Committee	9/21/22	Reviewed:	
	Medical Committees	Date	Revised:	
Endorsed:	Medical Executive Committee	11/22/22	Issued:	11/22/22
	Committees / Councils	Date		
Approved:	Josh Stripling, MD	9/21/22		
	Medical Director of Antimicrobial Stewardship	Date	Discontinued:	
Cross Ref:				

Guidelines for Transition from Intravenous to Oral or Long-Acting IV Antimicrobial Therapy in Select Patients with Certain Bacterial Infections

SYNOPSIS: Historically, the management of certain complex infections, including infective endocarditis, osteomyelitis, bacteremia and spinal epidural abscess, has included the use of several weeks of IV antibiotics, recent published studies have indicated the use of oral agents or long-acting IV antibiotics to complete therapy in these conditions is safe and effective, offering an alternative option for some patients. This guideline is not intended for use in conditions where oral antibiotic therapies are commonly used and IV therapies would not be indicated or appropriate treatment.

STRATEGY: Oral agents or long-acting IV antibiotics for the last few weeks of treatment for infections caused by bacterial organisms may be appropriate for patients who:

- Are in the medium- or high-risk IVAT category for discharge with a PICC or are in the low-risk IVAT category and have a non-addiction factor that may preclude discharge with PICC (e.g., homelessness)
- Are hospitalized for IV antimicrobial therapy but prefer to leave the hospital against medical advice and/or are interested or willing to receive oral or long-acting IV antibiotics as alternative treatment
- Would otherwise be discharged with a PICC but prefer an alternative option, for example:
    1. Out of concern for lack of access to follow-up for PICC removal
    2. Out of concern for difficulties receiving IV antibiotics post discharge, safety of IV line use, cost of IV antibiotics and cost of home health
    3. Out of concern for patients with altered mental status, dementia or other cognitive impairment where PICC therapy would introduce additional significant risk
    4. Others, as teams determine is best for patient-centered care

In transitioning a patient to oral or long-acting IV antimicrobial agents, clinical teams should ensure:

- The patient has improved clinically on current antibiotics.
- The patient is clinically and hemodynamically stable and otherwise ready for hospital discharge.
- Surgical or procedural source control has been achieved, if possible and indicated, and without persistent bacteremia.
- The patient can tolerate and has gastrointestinal function to absorb oral medications OR has no contraindication for long-acting IV antibiotic therapy.
- There are no psychosocial or logistical reasons to prefer IV therapy

Before transitioning to oral or long-acting IV antimicrobial agents:

- Patients should have an ID consult and the ID team must be involved in the decision-making process for consideration of these therapy options

➢ If addiction or substance use disorder is a factor in patient care, ensure Addiction Medicine has evaluated the patient, performed IVAT assessment, and provided recommendations. All patients with a substance use disorder should be linked to outpatient addiction treatments (via addiction medicine social work), and those with opioid use disorder should ideally be started and stable on a medication for opioid use disorder (i.e., buprenorphine-naloxone, extended-release naltrexone, or methadone dispensed at opioid treatment program).

Triggers for Implementation:
Criteria to consider in selecting patients who may be treated with oral agents or long-acting IV antibiotics:

1. **Oral antibiotics**
   a) Patients with infections requiring extended antibiotic therapy
   b) Cultures with only organisms that have shown susceptibility to oral agents with good activity against the pathogen and the infection being treated. In individual cases the ID consult team may also choose to use an oral regimen empirically if cultures remain negative.
   c) Patients who have stabilized, are afebrile, have no signs of worsening infection, and all necessary procedures prior to discharge have been completed (e.g., drainage of abscess or surgery)
   d) Repeated blood cultures are negative in those with infective endocarditis or *Staphylococcus aureus* bacteremia
   e) There is agreement that the patient will be able to take the medications (e.g., adherence, cost issues, etc.). If access to oral medications is a barrier, the medications will be supplied to the patient from a UAB retail pharmacy upon hospital discharge.
   f) As needed, refer to the document "Oral Antibiotic Guidelines for Common Organisms" for more detailed treatment considerations.

2. **Long-acting IV antibiotics (one dose of dalbavancin followed by discharge)**
   a) Patients with infections requiring extended antibiotic therapy
   b) Oral antibiotics are not active, appropriate, preferred, or an available option after discussion with the patient's care team
   c) Cultures with staphylococci, streptococci, enterococci, or other organisms susceptible to dalbavancin
   d) Patients who have stabilized, are afebrile, have no signs of worsening infection, and all necessary procedures prior to discharge have been completed (e.g., drainage of abscess or surgery)
   e) Repeated blood cultures are negative in those with infective endocarditis or *Staphylococcus aureus* bacteremia
   f) Patients with 1-2 weeks of planned IV therapy remaining for completion of treatment
   g) The use of oritavancin may also be considered in specific cases (e.g., VRE infection) for the last week of treatment if cultures are susceptible (oritavancin MIC ≤0.12 mcg/mL)

Patients who elope, leave AMA, or otherwise refuse IV antibiotic therapy:
There are occasions when the care team advises IV antibiotic therapy only, but the patient elopes, leaves against medical advice, or otherwise refuses/is noncompliant with the recommended IV therapy. In these cases, the physician may consider oral antibiotics or long-acting IV antibiotics even when all of the clinical criteria are not met considering the most benefit to the patient. In this case, it is important that the physician thoroughly explain that the oral or long-acting IV therapy is not recommended and may not be effective for the treatment of the patient's infection when prescribing. Risks should be thoroughly explained. Document informed refusal of the recommended therapy in the patient's medical record.

REFERENCES:

1. Oral antibiotic guidelines for common organisms. UAB Hospital Pharmacy and Therapeutics Committee Medication Guideline. Available at: UAB Antimicrobial Stewardship ONE Page and UAB Drug Formulary.
2. Wald-Dickler N, Holtom PD, Phillips MC, et al. Oral is the new IV. Challenging decades of blood and bone infection dogma: a systematic review. Am J Med 2022;135(3):369-79.
3. Baddour LM, Weimer MB, Wurcel AG, et al. Management of infective endocarditis in people who inject drugs: a scientific statement from the American Heart Association. Circulation 2022;146:00-00. doi: 10.1161/CIR.0000000000001090
4. Sikka MK, Gore S, Vega T, et al. "OPTIONS-DC", a feasible discharge planning conference to expand infection treatment options for people with substance use disorder. BMC Infect Dis 2021;21:772.
5. Rolfe RJ Jr, Mathews RE, Rodriguez JM, et al. Implementation of a standardized protocol for hospitalized patients who inject drugs and require long-term antibiotics reduces length of stay without increasing 30-day readmissions. Open Forum Infect Dis 2017;4(Suppl 1):S340–1. doi: 10.1093/ofid/ofx163.811.
6. Eaton EF, Mathews RE, Lane PS, et al. A 9-point risk assessment for patients who inject drugs and require intravenous antibiotics: focusing inpatient resources on patients at greatest risk of ongoing drug use. Clin Infect Dis. 2019;68(6):1041-1043. doi: 10.1093/cid/ciy722.

INTERDISCIPLINARY COLLABORATION

None	
Physician / Medical Committees<br>*Pharmacy and Therapeutics Committee*	Endorsement Date<br>*10/05/22*
Committees / Councils<br>*None*	Endorsement Date
Hospital Department(s)	Endorsement Date

Supersedes:	None
File Name:	Guidelines for Transition from Intravenous to Oral or Long-Acting IV Antimicrobial Therapy in Select Patients with Certain Bacterial Infections CG#136
REVISIONS: Consistent with Joint Commission Standards, this standard is to be reviewed **at least** every three years and/or as practice changes.	

Oral Antibiotic Guidelines for Common Organisms

Supplement to: Guidelines for Transition from Intravenous to Oral or Long-Acting IV Antimicrobial Therapy in Select Patients with Certain Bacterial Infections

When transitioning a patient to oral antimicrobial therapy, clinical teams should ensure:

- The isolated pathogen is susceptible in vitro to the antibiotic selected for treatment.
- The patient has improved clinically on current antibiotics.
- The patient is clinically and hemodynamically stable and otherwise ready for hospital discharge.
- Surgical or procedural source control has been achieved, if possible and indicated, and without persistent bacteremia.
- Repeated blood cultures are negative in those with infective endocarditis or *Staphylococcus aureus* bacteremia.
- The patient can tolerate and has gastrointestinal function to absorb oral medications.
- There are no psychosocial or logistical reasons to prefer IV therapy and there is agreement that the patient will be able to take the oral medications (e.g., adherence, cost issues, etc.). If access to oral medications is a barrier, the medications will be supplied to the patient from a UAB retail pharmacy upon hospital discharge.

**Endocarditis**	
**Pathogen**	**Oral Treatment Recommendations**
**Staphylococci**	
<u>Methicillin-Sensitive</u>[1,2-5]<br><br>*S. aureus* (MSSA)<br><br>Coagulase-negative staphylococci[a]	• Amoxicillin 1000 mg QID[e] + rifampin 600 mg BID[e] (penicillin susceptible only)<br>• Amoxicillin/clavulanate 875/125 mg[e] + rifampin 600 mg BID[e]<br>• Cefadroxil 1000 mg BID[e] + rifampin 600 mg BID[e]<br>• Cephalexin 500 mg QID[e] + rifampin 600 mg BID[e]<br>• Dicloxacillin 1000 mg QID + rifampin 600 mg BID[e]<br>• Linezolid 600 mg BID + rifampin 600 mg BID[e]<br>• Trimethoprim/sulfamethoxazole 800/160 mg 2 tablets BID[d,e]<br>• Levofloxacin 750 mg daily[e] + rifampin 600 mg BID[e]<br>• Ciprofloxacin 750 mg BID[e] + rifampin 600 mg BID[e]
<u>Methicillin-Resistant</u>[1,3,4]<br><br>*S. aureus* (MRSA)<br><br>Coagulase-negative staphylococci[a]	• Linezolid 600 mg BID + rifampin 600 mg BID[e]<br>• Trimethoprim/sulfamethoxazole 800/160 mg 2 tablets BID[d,e]
**Streptococci**[1,6]<br><br>Including beta-hemolytic[b] and viridans group streptococci[c])	Penicillin Susceptible (MIC ≤ 0.12 μg/mL)<br>• Amoxicillin 1000 mg QID[e] + rifampin 600 mg BID[e,f]<br>• Cefadroxil 1000 mg BID[e] + rifampin 600 mg BID[e]<br>• Linezolid 600 mg BID[f] + rifampin 600 mg BID[e]<br>• Linezolid 600 mg BID[f] + moxifloxacin 400 mg daily[f]<br><br>Penicillin Resistant (MIC > 0.12 μg/mL)<br>• Linezolid 600 mg BID + rifampin 600 mg BID[e]

	• Moxifloxacin 400 mg daily[f] + rifampin 600 mg BID[e]<br>• Moxifloxacin 400 mg daily[f] + clindamycin 600 TID
**Enterococci**[1]	• Linezolid 600 mg BID + rifampin 600 mg BID[e]<br>• Linezolid 600 mg BID + moxifloxacin 400 mg daily[f]<br>• Amoxicillin 1000 mg QID[e] + rifampin 600 mg BID[e]
**HACEK Group Gram-Negative Bacilli**[6]	
Haemophilus spp., *Aggregatibacter* spp., *Cardiobacterium hominis*, *Eikenella corrodens*, *Kingella* spp.	• Ciprofloxacin 500 mg BID[e,f]
**Bloodstream Infections**	
**Pathogen**	**Oral Treatment Recommendations**
**Staphylococci**	
<u>Methicillin-Sensitive</u>[7-9,10]<br><br>*S. aureus* (MSSA)<br><br>Coagulase-negative staphylococci	• Linezolid 600 mg BID<br>• Levofloxacin 750 mg daily[e,f]<br>• Trimethoprim/sulfamethoxazole 800/160 mg 2 tablets BID[d,e]<br>• Clindamycin 600 mg TID<br>• Cefadroxil 1000 mg BID[e]<br>• Cephalexin 500 mg QID[e]<br>• Dicloxacillin 1000 mg QID
<u>Methicillin-Resistant</u>[7-9]<br><br>*S. aureus* (MRSA)<br><br>Coagulase-negative staphylococci	• Linezolid 600 mg BID<br>• Trimethoprim/sulfamethoxazole 800/160 mg 2 tablets BID[d,e]
**Streptococci**[8,11]	
Beta-hemolytic Strep<br><br>Viridans group Strep<br><br>*S. pneumoniae* (Pneumococcus)	Penicillin Susceptible<br><br>• Amoxicillin 1000 mg BID[e]<br>• Cefpodoxime 400 mg BID[e]<br>• Linezolid 600 mg BID[f]<br>• Levofloxacin 750 mg daily[e,f]<br>• Moxifloxacin 400 mg daily[f]<br><br>Penicillin Resistant<br><br>• Linezolid 600 mg BID[f]<br>• Levofloxacin 750 mg daily[e,f]<br>• Moxifloxacin 400 mg daily[f]
**Enterococci**[9]	
Ampicillin-susceptible enterococci	• Amoxicillin 1000 mg BID[e]<br>• Linezolid 600 mg BID<br>• Levofloxacin 750 mg daily[e]

Ampicillin-resistant enterococci	• Linezolid 600 mg BID
**Gram Negative**[12,13]	
Enterobacterales	• Amoxicillin/clavulanate 875/125 mg BID[e]<br>• Cefadroxil 1000 mg BID[e]<br>• Cefpodoxime 400 mg BID[e]<br>• Ciprofloxacin 500 mg BID[e]<br>• Trimethoprim/sulfamethoxazole 800/160 mg BID[d,e]<br>• Moxifloxacin 400 mg daily[f]<br>• Levofloxacin 750 mg daily[e,f]
Pseudomonas aeruginosa	• Ciprofloxacin 750 mg BID[e]
Enterobacter cloacae, Citrobacter freundii, Klebsiella (previously *Enterobacter*) *aerogenes*	• Ciprofloxacin 500 mg BID[e]<br>• Trimethoprim/sulfamethoxazole 800/160 mg BID[d,e]<br>• Moxifloxacin 400 mg daily[f]<br>• Levofloxacin 750 mg daily[e,f]

**Osteomyelitis**[14,15]	
**Pathogen**	**Oral Treatment Recommendations**
**Staphylococci**	
Methicillin-Sensitive *S. aureus* (MSSA)[10,16,17]	• Levofloxacin 750 mg daily[b] + rifampin 600 mg daily<br>• Ciprofloxacin 500 mg BID[b] + rifampin 600 mg daily<br>• Trimethoprim/sulfamethoxazole 800/160 mg 2 tablets BID[a,b]<br>• Clindamycin 600 mg TID<br>• Linezolid 600 mg BID<br>• Doxycycline 100 mg BID<br>• Cefadroxil 1000 mg BID[b]
Methicillin-Resistant *S. aureus* (MRSA)[17]	• Trimethoprim/sulfamethoxazole 800/160 mg 2 tablets BID[a,b]<br>• Doxycycline 100 mg BID<br>• Linezolid 600 mg BID
Coagulase-negative staphylococci[10,16,17]	• Levofloxacin 750 mg daily[b] + rifampin 600 mg daily<br>• Trimethoprim/sulfamethoxazole 800/160 mg 2 tablets BID[a,b]<br>• Clindamycin 600 mg TID<br>• Linezolid 600 mg BID
**Streptococci**[8,11]	
Beta-hemolytic Strep<br>Viridans group Strep<br>*S. pneumoniae*	Penicillin Susceptible<br>• Amoxicillin 1000 mg BID[e]<br>• Cefpodoxime 400 mg BID[e]<br>• Linezolid 600 mg BID[f]<br>• Levofloxacin 750 mg daily[e]<br>• Moxifloxacin 400 mg daily[f]<br>Penicillin Resistant<br>• Linezolid 600 mg BID[f]

	• Levofloxacin 750 mg daily[e]<br>• Moxifloxacin 400 mg daily[f]
**Enterococci**[16]	
Ampicillin-susceptible enterococci	• Amoxicillin 1000 mg BID[e]<br>• Levofloxacin 750 mg daily[e]<br>• Linezolid 600 mg BID
Ampicillin-resistant enterococci	• Linezolid 600 mg BID
**Gram Negative**[16-19]	
Pseudomonas spp.	• Ciprofloxacin 750 mg BID[e]<br>• Levofloxacin 750 mg daily[e,f]
Enterobacter cloacae, *Citrobacter freundii*, *Klebsiella* (previously *Enterobacter*) *aerogenes*	• Ciprofloxacin 500 mg BID[e]<br>• Levofloxacin 750 mg daily[e,f]<br>• Trimethoprim/sulfamethoxazole 800/160 mg 2 tablets BID[d,e]
Other Enterobacterales	• Cefadroxil 1000 mg BID[e]<br>• Cefpodoxime 400 mg BID[e]<br>• Trimethoprim/sulfamethoxazole 800/160 mg 2 tablets BID[d,e]<br>• Doxycycline 100 mg BID<br>• Ciprofloxacin 500 mg BID[e]<br>• Levofloxacin 750 mg daily[e,f]
Rifampin[14]: Data on the benefit of adjunctive rifampin are mixed. The addition of rifampin in certain patients may be considered. Risks including side effects and potential drug interactions should be evaluated. Rifampin should never be used as monotherapy.	

**Prosthetic Joint Infections with Retained Prosthesis**[14,17,20]	
**Pathogen**	**Oral Treatment Recommendations**
**Staphylococci**	• Ciprofloxacin 500 BID[e] + rifampin 600 mg daily<br>• Levofloxacin 750 daily[e] + rifampin 600 mg daily<br>• Trimethoprim/sulfamethoxazole 800/160 mg 2 tablets BID[d,e] + rifampin 600 mg daily<br>• Doxycycline 100 mg BID + rifampin 600 mg daily<br>• Cefadroxil 1000 mg BID[e] + rifampin 600 mg daily
**Other organisms**	Treat with a pathogen-specific highly bioavailable oral antimicrobial therapy (see osteomyelitis recommendations above)

a. *S. epidermidis*, *S. lugdunensis*
b. *S. pyogenes*, *S. agalactiae* (group B Streptococcus)
c. *S. sanguis*, *S. mitis*, *S. salivarius*, *S. mutans*, *Gemella morbillorum* (formally *S. morbillorum*), *S. anginosus* group (*S.intermedius*, *S. anginosus*, *S. constellatus*)
d. Weight based dosing (7.5-10 mg/kg/day divided in 2 or 3 doses) can be considered in patients with total body weight ≥100 kg
e. Dose adjustments are recommended for patients with impaired renal function
f. Additional susceptibility testing may be necessary; breakpoints may not exist

References:

1. Iversen K, Ihlemann N, Gill SU, et al. Partial Oral versus Intravenous Antibiotic Treatment of Endocarditis. *N Engl J Med*. 2019;380(5):415-424.

2. Wildenthal JA, Atkinson A, Lewis S, et al. Outcomes of Partial Oral Antibiotic Treatment for Complicated S. aureus Bacteremia in People Who Inject Drugs. *Clin Infect Dis.* 2022.

3. Tissot-Dupont H, Gouriet F, Oliver L, et al. High-dose trimethoprim-sulfamethoxazole and clindamycin for Staphylococcus aureus endocarditis. *Int J Antimicrob Agents*. 2019;54(2):143-148.

4. Baddour LM, Weimer MB, Wurcel AG, et al. MaSnagement of Infective Endocarditis in People Who Inject Drugs: A Scientific Statement From the American Heart Association. *Circulation.* 2022;146.

5. Heldman AW, Hartert TV, Ray SC, et al. Oral Antibiotic Treatment of Right-sided Staphylococcal Endocarditis in Injection Drug Users: Prospective Randomized Comparison with Parenteral Therapy. *Am J Med.* 1996;101:68-76.

6. Baddour LM, Wilson WR, Bayer AS, et al. Infective Endocarditis in Adults: Diagnosis, Antimicrobial Therapy, and Management of Complications. *Circulation.* 2015;132:1435-1486.

7. Willekens R, Puig-Asensio M, Ruiz-Camps I, et al. Early Oral Switch to Linezolid for Low-risk Patients With Staph Bacteremia. *Clin Infect Dis*. 2019;69(3).

8. Wilcox M, Nathwani D, Dryden M. Linezolid compared with teicoplanin for the treatment of suspected or proven Gram-positive infections. *J Antimicrob Chemother.* 2004;53:335-344.

9. Wilcox MH, Tack KJ, Bouza E, et al. Complicated Skin and Skin-Structure Infections and Catheter-Related Bloodstream Infections: Noninferiority of Linezolid in a Phase 3 Study. *Clin Infect Dis.* 2009;48:203-12.

10. Schrenzel J, Harbarth S, Schockmel G, et al. A Randomized Clinical Trial to Compare Fleroxacin-Rifampicin with Flucloxacillin or Vancomycin for the Treatment of Staphylococcal Infection. *Clin Infect Dis.* 2004;39:1285-92.

11. San Pedro GS, Cammarata SK, Oliphant TH, Todisco T. Linezolid versus ceftriaxone/cefpodoxime in patients hospitalized for the treatment of Streptococcus pneumoniae pneumonia. *Scand J Infect Dis*. 2002;34(10):720-728.

12. Park TY, Choi JS, Song TJ, Do JH, Choi SH, Oh HC. Early Oral Antibiotic Switch Compared with Conventional Intravenous Antibiotic Therapy for Acute Cholangitis with Bacteremia. *Dig Sci*. 2014;59:2790-2796.

13. Sutton JD, Stevens VW, Chang NCN, Khader K, Timbrook TT, Spivak ES. β-Lactam vs Fluoroquinolones or Trimethoprim-Sulfamethoxazole for Enterobacterales Bacteremia From a Urine Source Study. *JAMA Netw Open*. 2020;3(10).

14. Spellberg B, Aggrey G, Brennan MB, et al. Use of Novel Strategies to Develop Guidelines for Management of Pyogenic Osteomyelitis in Adults: A WikiGuidelines Group Consensus Statement. *JAMA Netw Open*. 2020;5(5).

15. Li HK, Rombach I, Zambellas R, et al. Oral versus Intravenous Antibiotics for Bone and Joint Infection. *N Engl J Med*. 2019;380(5):425-436.

16. Gentry LO, Rodriguez-Gomez G. Ofloxacin versus parenteral therapy for chronic osteomyelitis. *Antimicrob Agents Chemother*. 1991;35(3):538-541.

17. Euba G, Murillo O, Fernandez-Sabe N, et al. Long-Term Follow-Up Trial of Oral Rifampin-Cotrimoxazole Combination versus Intravenous Cloxacillin in Treatment of Chronic Staphylococcal Osteomyelitis. *Antimicrob Agents Chemother.* 2009;53(6):2672-2676.

18. Gentry LO, Rodriguez GG. Oral Ciprofloxacin Compared with Parenteral Antibiotics in the Treatment of Osteomyelitis. *Antimicrob Agents Chemother.* 1990;34(1):40-43.

19. Mader JT, Cantrell JS, Calhoun J. Oral ciprofloxacin compared with standard parenteral antibiotic therapy for chronic osteomyelitis in adults. *J Bone Joint Surg Am*. 1990;72(1):104-110.

20. Osmon DR, Berbari EF, Berendt AR, et al. Diagnosis and Management of Prosthetic Joint Infection: Clinical Practice Guidelines by the Infectious Diseases Society of Americaa. *Clin Infect Dis.* 2013;56(1).

Supplemental Material 2: ICD-10 Codes

Endocarditis

I38	I33.9	I33.0	B37.6	I39

Bacteremia_Sepsis

A41.2	A41.01	A41.1	A41.02	A41.51	A41.4
A41.50	A41.3	A41.52	A41.53	A41.59	A41.89
I26.90	I40.0	I76	R65.21	R78.81	R65.20
A42.7	A40.3	A41.9	A40.9	B37.7	R57.8

Osteomyelitis of bone/spine

H05.021	H05.022	H05.023	H05.029	M46.20	M46.28
M86.00	M86.011	M86.012	M86.019	M86.021	M86.022
M86.029	M86.031	M86.032	M86.039	M86.041	M86.042
M86.049	M86.051	M86.052	M86.059	M86.061	M86.062
M86.069	M86.071	M86.072	M86.079	M86.08	M86.09
M86.10	M86.111	M86.112	M86.119	M86.121	M86.122
M86.129	M86.131	M86.132	M86.139	M86.141	M86.142
M86.149	M86.151	M86.152	M86.159	M86.161	M86.162
M86.169	M86.171	M86.172	M86.179	M86.18	M86.19
M86.20	M86.211	M86.212	M86.219	M86.221	M86.222
M86.229	M86.231	M86.232	M86.239	M86.241	M86.242
M86.249	M86.251	M86.252	M86.259	M86.261	M86.262
M86.269	M86.271	M86.272	M86.279	M86.28	M86.29
M86.30	M86.311	M86.312	M86.319	M86.321	M86.322
M86.329	M86.331	M86.332	M86.339	M86.341	M86.342
M86.349	M86.351	M86.352	M86.359	M86.361	M86.362
M86.369	M86.371	M86.372	M86.379	M86.38	M86.39
M86.40	M86.411	M86.412	M86.419	M86.421	M86.422
M86.429	M86.431	M86.432	M86.439	M86.441	M86.442
M86.449	M86.451	M86.452	M86.459	M86.461	M86.462
M86.469	M86.471	M86.472	M86.479	M86.48	M86.49
M86.50	M86.511	M86.512	M86.519	M86.521	M86.522
M86.529	M86.531	M86.532	M86.539	M86.541	M86.542
M86.549	M86.551	M86.552	M86.559	M86.561	M86.562
M86.569	M86.571	M86.572	M86.579	M86.58	M86.59
M86.60	M86.611	M86.612	M86.619	M86.621	M86.622
M86.629	M86.631	M86.632	M86.639	M86.641	M86.642
M86.649	M86.651	M86.652	M86.659	M86.661	M86.662
M86.669	M86.671	M86.672	M86.679	M86.68	M86.69
M86.8X0	M86.8X1	M86.8X2	M86.8X3	M86.8X4	M86.8X5
M86.8X6	M86.8X7	M86.8X8	M86.8X9	M86.9	G04.89
G04.91	G05.4				

Septic Arthritis

M00	M00.0	M00.00	M00.01	M00.011	M00.012
M00.019	M00.02	M00.021	M00.022	M00.029	M00.03
M00.031	M00.032	M00.039	M00.04	M00.041	M00.042
M00.049	M00.05	M00.051	M00.052	M00.059	M00.06
M00.061	M00.062	M00.069	M00.07	M00.071	M00.072
M00.079	M00.08	M00.09	M00.1	M00.10	M00.11
M00.111	M00.112	M00.119	M00.12	M00.121	M00.122

M00.129	M00.13	M00.131	M00.132	M00.139	M00.14
M00.141	M00.142	M00.149	M00.15	M00.151	M00.152
M00.159	M00.16	M00.161	M00.162	M00.169	M00.17
M00.171	M00.172	M00.179	M00.18	M00.19	M00.2
M00.20	M00.21	M00.211	M00.212	M00.219	M00.22
M00.221	M00.222	M00.229	M00.23	M00.231	M00.232
M00.239	M00.24	M00.241	M00.242	M00.249	M00.25
M00.251	M00.252	M00.259	M00.26	M00.261	M00.262
M00.269	M00.27	M00.271	M00.272	M00.279	M00.28
M00.29	M00.8	M00.80	M00.81	M00.811	M00.812
M00.819	M00.82	M00.821	M00.822	M00.829	M00.83
M00.831	M00.832	M00.839	M00.84	M00.841	M00.842
M00.849	M00.85	M00.851	M00.852	M00.859	M00.86
M00.861	M00.862	M00.869	M00.87	M00.871	M00.872
M00.879	M00.88	M00.89	M00.9	M01	M01.X
M01.X0	M01.X1	M01.X11	M01.X12	M01.X19	M01.X2
M01.X21	M01.X22	M01.X29	M01.X3	M01.X31	M01.X32
M01.X39	M01.X4	M01.X41	M01.X42	M01.X49	M01.X5
M01.X51	M01.X52	M01.X59	M01.X6	M01.X61	M01.X62
M01.X69	M01.X7	M01.X71	M01.X72	M01.X79	M01.X8
M01.X9					

Prosthetic Joint Infection

T84.50	T84.5-

Hardware-associated osteomyelitis

T84.60	T84.61	T84.610	T84.611	T84.612	T84.613
T84.614	T84.615	T84.619	T84.62	T84.620	T84.621
T84.622	T84.623	T84.624	T84.625	T84.629	T84.63
T84.69					

Supplemental Material 3: REDCap Abstraction Forms

Linking Document

Record ID

____

Record_ID from final output. This is the crosswalk ID.

____

MRN

____

Admission Date

____

Basic Clinical Data Abstraction

Patient Initials	__________________________________<br>(First and last initial as displayed at top left corner of chart. (e.g. John Smith = J.S.))
Housing status	◯ Housed<br>◯ Unhoused<br>◯ Unknown/Unclear<br>(Housing status as recorded in EHR demographics or social work documentation pertaining to the encounter.)
What was the discharge type?	◯ Routine<br>◯ Against medical advice (AMA) or patient-directed (PDD)<br>◯ Death<br>(Discharge type as noted in discharge summary, discharge case management/social work notes, or other documentation pertaining to discharge planning.)
Discharge disposition	◯ Home<br>◯ To skilled nursing facility<br>◯ To street<br>◯ To shelter<br>◯ To rehab<br>◯ Other (specify)<br>◯ Unknown/unclear<br>◯ Died during encounter<br>(Discharge disposition as noted in discharge summary, discharge case management/social work notes, or other documentation pertaining to discharge planning.)
What was the discharge disposition?	__________________________________

Note that the following questions are encounter-specific. That is, you should NOT look at documentation outside of the specific encounter to obtain the answers to the questions.

Also, only certain note types are to be evaluated for this information, and this should only be abstracted from the HPI and A+P sections. They are as follows:

- Primary team H+P
- ID consult H+P
- Any consult team H+P's INCLUDING addiction medicine
- Be sure to use the structured text from addiction medicine for any substance type questions
- Discharge summary (one liner and problem list only)

Does the patient have noted PAST illicit substance use BESIDES alcohol and tobacco (i.e. stimulants, opioids, etc.)?	◯ No<br>◯ Yes<br>(As established by review of primary team H+P, ID consult H+P, addiction medicine consult H+P (if present), and discharge summary problem lists and "one liners.")

History of use of which substances?	☐ Cannabis<br>☐ Cocaine<br>☐ Amphetamine-type stimulants<br>☐ Inhalants<br>☐ Sedatives or Sleeping Pills<br>☐ Hallucinogens<br>☐ Opioids<br>☐ Other (specify)<br>(As established by review of primary team H+P, ID consult H+P, addiction medicine consult H+P (if present), and discharge summary problem lists and "one liners.")
What substance(s)?	__________________________________
Does the patient have a history of current or past injection drug use?	○ No<br>○ Yes<br>(As established by review of primary team H+P, ID consult H+P, addiction medicine consult H+P (if present), and discharge summary problem lists and "one liners.")
Does the patient have noted CURRENT, ACTIVE illicit substance use BESIDES alcohol and tobacco (i.e. stimulants, opioids, etc.)?	○ No<br>○ Yes<br>(As established by review of primary team H+P, ID consult H+P, addiction medicine consult H+P (if present), and discharge summary problem lists and "one liners.")
Active use of which substances?	☐ Cannabis<br>☐ Cocaine<br>☐ Amphetamine-type stimulants<br>☐ Inhalants<br>☐ Sedatives or Sleeping Pills<br>☐ Hallucinogens<br>☐ Opioids<br>☐ Other (specify)<br>(As established by review of primary team H+P, ID consult H+P, addiction medicine consult H+P (if present), and discharge summary problem lists and "one liners.")
What substance(s)?	__________________________________
Does the patient have a noted history of any psychiatric illness?	○ No<br>○ Yes<br>(As established by review of primary team H+P, consult team H+Ps, and discharge summary problem lists as well as "one liners.")

History of which psychiatric illness(es)?	☐ Depression<br>☐ Anxiety<br>☐ MDD<br>☐ GAD<br>☐ Schizophrenia, all types<br>☐ Bipolar disorder<br>☐ Substance induced psychotic disorder<br>☐ Personality disorder<br>☐ Schizoaffective disorder<br>☐ Other (please specify)
What disorder(s)?	__________________________________
Was the patient readmitted to the hospital within 3 months of discharge?	◯ No<br>◯ Yes<br>(As established by presence of an admission H+P document within 90 days of discharge summary.)

ID Abstraction

What are the patient's first and last initials	__________________________________<br>(First and last initial as displayed at top left corner of chart. (e.g. John Smith = J.S.))
What is the primary bacterial infection diagnosis category or categories?	☐ Bacteremia<br>☐ Osteomyelitis<br>☐ Septic arthritis<br>☐ Native valve endocarditis<br>(As established by review of ID notes.)
What were the pathogen(s) causing bacteremia?	☐ MSSA<br>☐ MRSA<br>☐ Streptoccoci (or nutritionally variant strep)<br>☐ Enterococci<br>☐ HACEK organism<br>☐ Gram negative organism<br>☐ Other<br>(As established by review of ID notes and culture reports)
What were the pathogen(s) causing bacteremia?	__________________________________<br>(Separate by semicolons)
Did microbiologic failure of BACTEREMIA treatment occur?	○ No<br>○ Yes<br>(Defined as growth of at least one the index organism(s) (not necessarily same susceptibilities) from blood within 30 days of completion of prescribed antibiotic therapy. Only count culture data reported in the UAB "micro" tab or explicitly noted in the infectious diseases consult note(s) from subsequent encounters.)
What were the pathogen(s) causing osteomyelitis?	☐ MSSA<br>☐ MRSA<br>☐ Streptoccoci (or nutritionally variant strep)<br>☐ Enterococci<br>☐ HACEK organism<br>☐ Gram negative organism<br>☐ Other<br>(As established by review of ID notes and culture reports)
What were the pathogen(s) causing osteomyelitis?	__________________________________<br>(Separate by semicolons)
Was hardware involved in the osteomyelitis?	○ No<br>○ Yes<br>(As established by review of ID and relevant subspecialty notes/op reports)
What was the status of the osteomyelitis-related infected hardware at discharge?	○ Removed<br>○ Retained<br>○ Partially retained<br>○ Unknown/Unable to determine

Was this acute (28 or fewer days from implantation) or chronic (>28 days from implantation) hardware-associated osteomyelitis?	◯ Acute<br>◯ Chronic<br>◯ Unknown
Did microbiologic failure of OSTEOMYELITIS treatment occur?	◯ No<br>◯ Yes<br>(Defined as isolation of at least one of the index organism(s) (not necessarily same susceptibilities) from same site from at least one sterile site or sinus tract culture within 90 days of completion of prescribed antibiotic therapy. Only count culture data reported in the UAB "micro" tab or explicitly noted in the infectious diseases consult note(s) from subsequent encounters.)
What were the pathogen(s) causing septic arthritis?	☐ MSSA<br>☐ MRSA<br>☐ Streptoccoci (or nutritionally variant strep)<br>☐ Enterococci<br>☐ HACEK organism<br>☐ Gram negative organism<br>☐ Other<br>(As established by review of ID notes and culture reports)
What were the pathogen(s) causing septic arthritis?	________________________________<br>(Separate by semicolons)
Was hardware involved in the septic arthritis?	◯ No<br>◯ Yes<br>(As established by review of ID and relevant subspecialty notes/op reports)
What was the status of the septic arthritis-related infected hardware at discharge?	◯ Removed<br>◯ Retained<br>◯ Partially retained<br>◯ Unknown/Unable to determine
Was this an acute (28 or fewer days from implantation) or chronic (>28 days from implantation) PJI?	◯ Acute<br>◯ Chronic<br>◯ Unknown
Did microbiologic failure of SEPTIC ARTHRITIS treatment occur?	◯ No<br>◯ Yes<br>(Defined as isolation of at least one of the index organism(s) (not necessarily same susceptibilities) from same site from at least one sterile site or sinus tract culture within 90 days of completion of prescribed antibiotic therapy. Only count culture data reported in the UAB "micro" tab or explicitly noted in the infectious diseases consult note(s) from subsequent encounters.)

What were the pathogen(s) causing native valve endocarditis?	☐ MSSA<br>☐ MRSA<br>☐ Streptococci (or nutritionally variant strep)<br>☐ Enterococci<br>☐ HACEK organism<br>☐ Gram negative organism<br>☐ Other<br>(As established by review of ID notes and culture reports)
What were the pathogen(s) causing native valve endocarditis?	__________________________________<br>(Separate by semicolons)
What valve(s) were involved?	☐ Tricuspid<br>☐ Pulmonic<br>☐ Mitral<br>☐ Aortic<br>☐ Other<br>(As established by review of ID, cardiology, and cardiac surgery note(s))
Was a pacemaker or other cardiac device present?	○ No<br>○ Yes
Was the pacer or other cardiac device involved in the infection?	○ No<br>○ Yes
What was the status of the infected pacemaker or other cardiac hardware at discharge?	○ Removed<br>○ Retained<br>○ Partially retained<br>○ Unknown/Unable to determine
Did microbiologic failure of NATIVE VALVE ENDOCARDITIS treatment occur?	○ No<br>○ Yes<br>(Defined as isolation of at least one of the index organism(s) (not necessarily same susceptibilities) from same site from at least one sterile site or sinus tract culture within 90 days of completion of prescribed antibiotic therapy. Only count culture data reported in the UAB "micro" tab or explicitly noted in the infectious diseases consult note(s) from subsequent encounters.)
For how many calendar days were IV antibiotics administered during the inpatient stay?	__________________________________<br>(As established by viewing the MAR or ID Summary/equivalent.)
What type of antibiotic treatment was prescribed on discharge?	☐ No antibiotics<br>☐ Oral antibiotics<br>☐ Long-acting IV antibiotics<br>☐ IV antibiotics via OPAT<br>☐ Other antibiotic option<br>(As established by review of ID signoff note, discharge orders, discharge summary, and other relevant documentation.)

What was the other antibiotic option prescribed?	__________________________________ (Note that combinations of IV and oral should be indicated by checking multiple boxes in the above question, not put here.)
After discharge, is there any indication that antibiotics stopped or changed for an adverse drug event or suspected adverse drug event?	◯ No<br>◯ Yes<br>(Based on review of OPAT documentation, clinic follow-up notes, telephone encounters, portal messages, and other documentation for the duration of the prescribed antibiotic treatment.)
What ADE or ADEs led to discontinuation?	☐ Anaphylaxis<br>☐ Acute kidney injury<br>☐ Acute liver injury<br>☐ Other allergic reaction besides anaphylaxis (AIN, skin rash, etc.)<br>☐ Serious cutaneous adverse reaction (SCAR)<br>☐ Other non-SCAR non-allergy ADR<br>☐ GI intolerance<br>☐ Other intolerance/ADR<br>☐ Unknown/unable to determine<br>(Based on review of OPAT documentation, clinic follow-up notes, telephone encounters, portal messages, and other documentation for the duration of the prescribed antibiotic treatment.)
After discharge, is there any indication that OPAT therapy was stopped for an adverse event related to vascular access?	◯ No<br>◯ Yes<br>(Based on review of OPAT documentation, clinic follow-up notes, telephone encounters, portal messages, and other documentation for the duration of the prescribed antibiotic treatment.)
Did the vascular access event result in readmission?	◯ No<br>◯ Yes
Did the vascular access event result in death?	◯ No<br>◯ Yes
